# Measures of Global Health Status on Dialysis Signal Early Rehospitalization Risk after Kidney Transplantation

**DOI:** 10.1371/journal.pone.0156532

**Published:** 2016-06-03

**Authors:** Meera N. Harhay, Alexander S. Hill, Wei Wang, Orit Even-Shoshan, Adam S. Mussell, Roy D. Bloom, Harold I. Feldman, Jason H. Karlawish, Jeffrey H. Silber, Peter P. Reese

**Affiliations:** 1 Division of Nephrology, Department of Medicine, Drexel University College of Medicine, Philadelphia, Pennsylvania, United States of America; 2 Department of Pediatrics, The Children’s Hospital of Philadelphia, Philadelphia, Pennsylvania, United States of America; 3 Department of Surgical Outcomes and Analysis, Kaiser Permanente, San Diego, California, United States of America; 4 University of Pennsylvania, Department of Biostatistics and Epidemiology, Perelman School of Medicine at the University of Pennsylvania, Philadelphia, Pennsylvania, United States of America; 5 Renal Electrolyte & Hypertension Division, Perelman School of Medicine at the University of Pennsylvania, Philadelphia, Pennsylvania, United States of America; 6 Division of Geriatrics, Perelman School of Medicine at the University of Pennsylvania, Philadelphia, Pennsylvania, United States of America; University of Utah School of Medicine, UNITED STATES

## Abstract

**Background:**

Early rehospitalization (<30 days) after discharge from kidney transplantation (KT) is associated with poor outcomes. We explored summary metrics of pre-transplant health status that may improve the identification of KT recipients at risk for early rehospitalization and mortality after transplant.

**Materials and Methods:**

We performed a retrospective cohort study of 8,870 adult (≥ 18 years) patients on hemodialysis who received KT between 2000 and 2010 at United States transplant centers. We linked Medicare data to United Network for Organ Sharing data and data from a national dialysis provider to examine pre-KT (1) Elixhauser Comorbidity Index, (2) physical function (PF) measured by the Short Form 36 Health Survey, and (3) the number of hospitalizations during the 12 months before KT as potential predictors of early rehospitalization after KT. We also explored whether these metrics are confounders of the known association between early rehospitalization and post-transplant mortality.

**Results:**

The median age was 52 years (interquartile range [IQR] 41, 60) and 63% were male. 29% were rehospitalized in <30 days, and 20% died during a median follow-up time of five years (IQR 3.6–6.5). In a multivariable logistic model, kidney recipients with more pre-KT Elixhauser comorbidities (adjusted odds ratio [aOR] 1.09 per comorbidity, 95% Confidence Interval [CI] 1.07–1.11), the poorest pre-KT PF (aOR 1.24, 95% CI 1.08–1.43), or >1 pre-KT hospitalizations (aOR 1.32, 95% CI 1.17–1.49) were more likely to be rehospitalized. All three health status metrics and early rehospitalization were independently associated with post-KT mortality in a multivariable Cox model (adjusted hazard ratio for rehospitalization: 1.41, 95% CI 1.28–1.56)

**Conclusions:**

Pre-transplant metrics of health status, measured by dialysis providers or administrative data, are independently associated with early rehospitalization and mortality risk after KT. Transplant providers may consider utilizing metrics of pre-KT global health status as early signals of vulnerability when transitioning care after KT.

## Introduction

Kidney transplant recipients commonly experience early rehospitalization events, defined as hospitalizations occurring less than 30 days after discharge from kidney transplantation (KT). These events have come under increasing scrutiny given their diverse causes and high costs [1–4], and studies that have shown that patients who are rehospitalized early after KT are at higher risk of late rehospitalizations and death [2, 5, 6]. The association of early rehospitalization after KT and poor long-term outcomes may be partially explained by recent evidence that many early rehospitalization events are related to patients’ pre-transplant health status, which, in turn, is strongly and negatively influenced by prolonged exposure to dialysis therapy [2, 7–14]. Unfortunately, the need for prolonged dialysis therapy prior to KT is a reality for many KT recipients, as the short supply of available organs for transplant has resulted in long waiting times for KT and fewer pre-emptive transplantations [15, 16].

In the United States (US), in response to knowledge of the deleterious health effects of prolonged dialysis therapy and disparities in timely referral for KT, recent changes in organ allocation policy have provided retroactive waiting time accrual from the start of dialysis therapy for all patients on the transplant waiting list[17, 18]. As a result of these policy initiatives, more patients with numerous years of dialysis exposure are expected to receive KT[18]. Therefore, transplant providers are in greater need of tools that provide insight on the burden of poor health status among dialysis recipients who receive KT, and the implications of poor health at the time of KT on the risk of early rehospitalization and other adverse post-transplant outcomes.

Several commonly used summary measures of pre-transplant health may have utility in assessing rehospitalization risk after KT. Comorbidity scores using administrative data such as the Elixhauser Comorbidity Index [19] have been validated to enable comparisons of outcomes between hospitals, including transplant centers, with diverse patient populations [20–25]. The Elixhauser Index has merits as a metric of rehospitalization risk as it reflects not only the burden of major comorbidities but also receipt of health services for comorbid conditions. A 2009 study using Medicare data alone found that kidney recipients with greater than two Elixhauser diagnoses prior to KT were more likely to be rehospitalized early after KT [26]. However, it is unknown if the Elixhauser Index provides unique insight into early rehospitalization risk after KT when considering transplant factors not captured in Medicare, including waiting time and graft quality.

Two alternative summary measures of global health that may be available to KT providers are self-reported assessment of physical function (PF) and health care utilization patterns pre-KT. Dialysis providers have been urged to screen PF regularly per the Kidney Disease Quality Initiative Guidelines; the PF domain of the Short Form 36 (SF-36) is a validated instrument to serve this purpose [27–31]. However, despite a growing interest in functional metrics to identify at-risk dialysis and KT patients [7, 10, 32–35], no studies to date have explored the PF domain of the SF-36 as a predictor of early rehospitalization after KT. Also, dialysis patients have high rates of health care utilization, including acute hospitalizations [36]. Prior hospitalizations are strongly associated with early rehospitalization in general medicine patients [37], but to date, these data have not yet been integrated into studies of early rehospitalization after KT.

Therefore, the central objectives of this study were to compare the associations of three summary assessments of pre-transplant health status to the outcome of early rehospitalization after KT, and to assess the effect of adjustment for these health status metrics on the known association between early rehospitalization after KT and mortality. In a national cohort of dialysis patients who received KT, we compared models for early rehospitalization that utilized traditional patient, donor, and process-related risk factors with the addition of: 1) the Elixhauser Comorbidity Index, 2) PF obtained from the SF-36 instrument, and 3) the number of hospitalizations in one year prior to KT. We also examined whether pre-transplant health status is an important confounder of the known association between early rehospitalization after KT and death.

## Materials and Methods

### Study Design

We performed a retrospective cohort study of adult (≥18 years at wait-listing) dialysis recipients who received KT in the United States using a linked dataset from three organizations: the United Network for Organ Sharing (UNOS)/Organ Procurement and Transplantation Network (OPTN); Fresenius Medical Care, a national provider of chronic dialysis services that provided service for 33% of all dialysis patients in the US during the study period [38]; and the Centers for Medicare and Medicaid Services. The primary outcome was early rehospitalization, defined as readmission to an acute care facility within 30 days of discharge from KT. The secondary outcomes were: 1) a composite outcome of early mortality or early rehospitalization, and 2) mortality after KT. The primary exposures were recipients’ 1) pre-transplant Elixhauser Comorbidity Index, 2) score on the PF subscale of the SF-36 instrument, and 3) number of prior hospitalizations to an acute care facility in 12 months before transplant.

### Ethics Statement

The work described was approved by the University of Pennsylvania Institutional Review Board, which approved a waiver of authorization for retrospective review of existing medical record data. Subject records were de-identified prior to analysis. The clinical and research activities being reported are consistent with the Principles of the Declaration of Istanbul as outlined in the ‘Declaration of Istanbul on Organ Trafficking and Transplant Tourism.’

### Study Cohort

**Fig 1:**
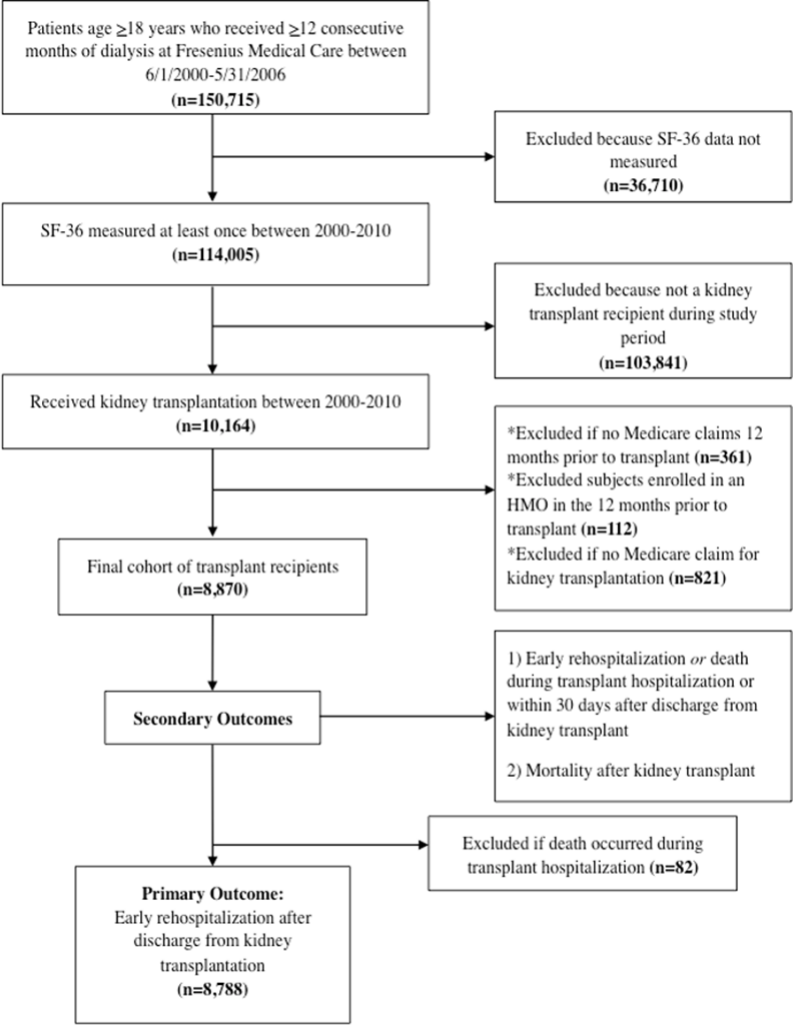
Participant Inclusion Flow Diagram.

The study cohort was derived from patients who received ≥12 months of chronic dialysis provided by Fresenius Medical Care, and had completed all 10 questions of the PF scale of the SF36 at least once during the observation period (Fig 1) [34]. Patients were included if they received KT between 2000 and 2010. In order to assure that study subjects were Medicare beneficiaries prior to KT, patients were excluded if they had no Medicare claims in the 12 months prior to transplant (n = 361), if they were enrolled in a health maintenance organization in the 12 months prior to KT (n = 112), and if they had no Medicare claim for their transplant admission (n = 821). For the primary outcome analysis, patients who died before discharge from KT (n = 82) were excluded. These subjects were included in the secondary analysis of early death or rehospitalization as a composite outcome, and in the analysis of post-KT mortality.

### Data Sources

The UNOS/OPTN dataset provided demographic and clinical information for each KT recipient as well as donor data; these data were reported by the transplant center at the time of addition to the waiting list and transplantation [39]. Recipients’ pre-transplant Elixhauser Comorbidity Index was calculated using Medicare International Classification of Disease codes [19]. We included all available Medicare claims data from 180 days prior to the recipient waiting list date. We excluded codes for renal failure from the Elixhauser calculation, as all subjects were KT recipients [26]. The SF-36 instrument was administered, by protocol, to patients annually at Fresenius dialysis centers. We included the PF score collected closest in time prior to the date of KT. The PF scale consists of 10 questions that assess patients’ self-reported challenges completing common physical activities requiring varying levels of effort, such as bending and kneeling or walking a mile. PF scores were transformed into a scale from 0 to 100 [40], and tested as a categorical variable based on quartiles. Prior health care utilization, derived from Medicare claims, was defined as the number of prior hospitalizations to an acute care facility in 12 months prior to KT. Our primary outcome, early rehospitalization to an acute care facility after KT, was ascertained using Medicare data. Mortality data for our secondary outcomes was ascertained from the OPTN, which receives regular updated mortality data from transplant centers and the Social Security Death Master File.

All analyses were performed using SAS (version 9.3, 2011) and R (2014): A language and environment for statistical computing (Vienna, Austria). Categorical variables (e.g., gender, race) were described by their frequencies. Continuous variables (e.g., years on dialysis) were described by their mean, median, range, and standard deviation. Binary variables were compared between groups using the chi-square test or Fisher’s exact test, as appropriate. Continuous variables were compared based on their distributions.

### Model Building Strategy

For our primary outcome, we used a modeling approach with the goal of defining the optimal permutation of pre-transplant health metrics that explain early rehospitalization risk. First, we generated a baseline logistic regression model of early rehospitalization using traditional recipient, donor, and center risk factors [1, 2, 6, 8, 21, 41–48]. We compared our baseline model to models in which we added the Elixhauser Comorbidity Index (model 1), PF assessment (model 2), prior hospitalizations (model 3), Elixhauser + PF (model 4), Elixhauser + prior hospitalizations (model 5), PF + prior hospitalizations (model 6) and all three metrics (model 7).

### Secondary Analysis of a Composite Outcome: Early Mortality or Early Rehospitalization

We fit logistic regression models for the composite outcome of early mortality, defined as death within the transplant hospitalization or within 30 days of discharge from KT, or early rehospitalization. We compared multivariable models utilizing the three global health metrics in a method analogous to the primary outcome, detailed above.

### Secondary Analysis: Post-Transplant Mortality

We fit a Cox Proportional Hazard model to examine the unadjusted association of early rehospitalization as a time-dependent covariate with post-KT mortality (model A). We compared this model to a Cox model adjusted for traditional covariates alone (model B), and to models that were adjusted for rehospitalization (as a time-dependent covariate), traditional risk factors, and iterations of the global health metrics under study (models C-I and a fully adjusted model). We compared model fit and predictive ability of all models. Subjects were censored if they were alive at the end of the follow-up period, July 31, 2010. We confirmed the proportional hazards assumption with visual inspection of log-log plots.

### Comparison of Models–Explaining versus Predicting Post-Transplant Outcomes

For our primary and secondary outcomes, we first aimed to identify the models requiring a minimum of additional data that also best explained early rehospitalization risk. To identify the best explanatory model, we compared our expanded models to the baseline model based on the Akaike Information Criterion (AIC), which is an assessment of model fit that penalizes models with additional covariates [49]. Second, to examine the ability of pre-transplant health metrics to improve prediction of early rehospitalization and our secondary outcomes, we calculated and compared c-statistics corresponding to our baseline and expanded models [49–51]. Also, since comorbidities, PF, and health care utilization are likely interrelated, we calculated variance inflation factors (VIF) for the three metrics in the fully adjusted rehospitalization model [52].

### Covariates

The following variables were considered traditional risk factors [1, 2, 6, 8, 21, 41–48] in our multivariable models: (1) *recipient* age category at transplant, sex, race, hepatitis C serostatus, obesity by body mass index (≥30 kg/m^2^), dialysis vintage (years), time on the waitlist (years), history of diabetes, history of previous solid organ transplant, education status, (2) *donor* type (live vs. deceased donor, expanded criteria deceased [ECD] donor, defined as donor age >60 years, or >50 years with comorbid conditions [53]); (3) *allograft* variables of delayed graft function, and (4) *process-of-care* variables of length of initial transplant hospitalization (days), weekend discharge (defined as discharge on Saturday or Sunday), and low transplant center volume (defined as <150 kidney transplants performed, on average, per year).

### Missing data and Sensitivity Analyses

Less than 0.1% of the cohort had missing data for any covariate, with the exception of body mass index (missing in 12.39%) and education status (missing in 17.11%). We performed sensitivity analyses in which individuals with missing data on body mass index and education status were first assigned to the lowest category then to the highest category, respectively. For the final analyses, we performed multiple imputation [54] to generate predicted values of body mass index and education status for those individuals with missing data.

## Results

### Patient Characteristics

**Fig 2:**
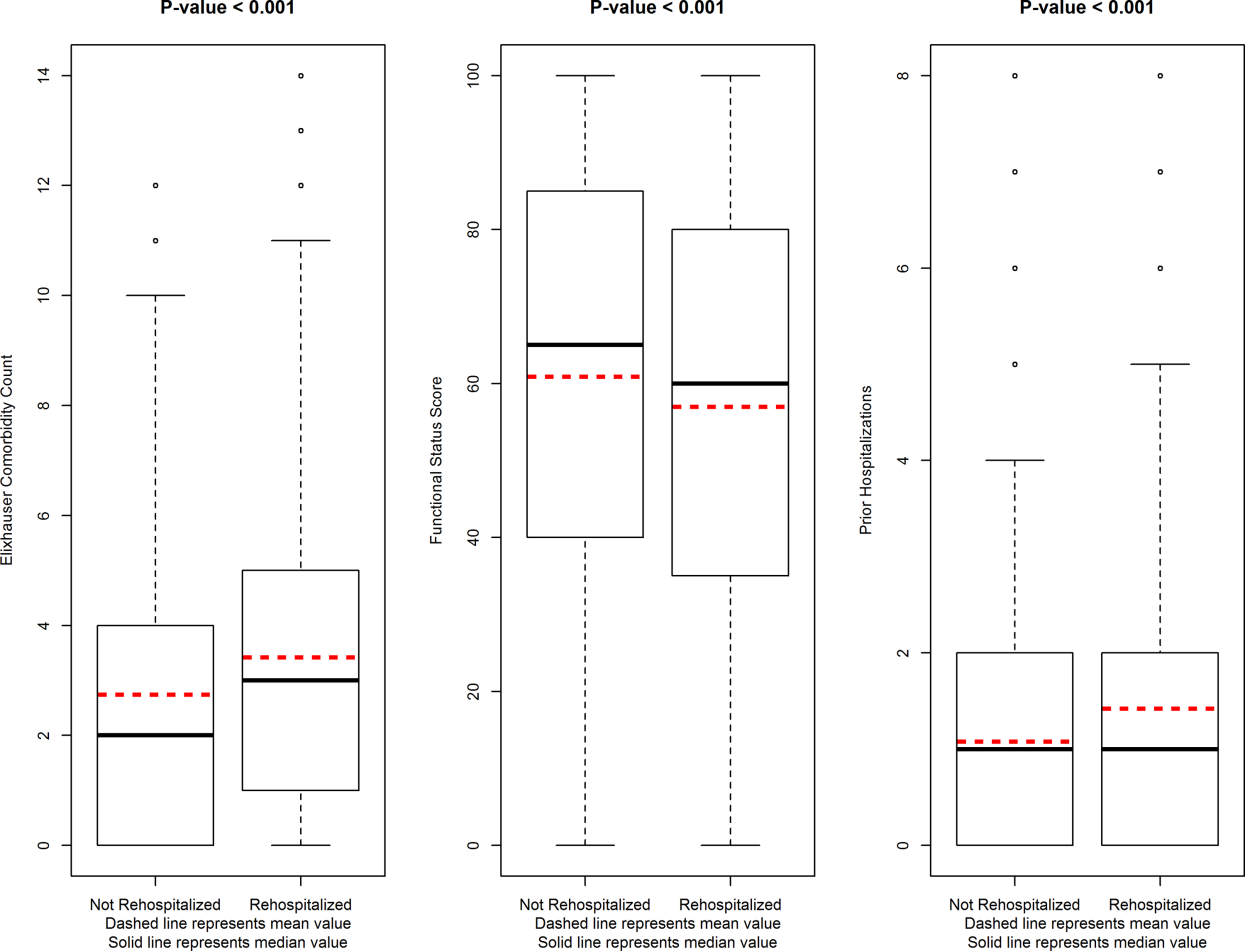
Distribution of Health Metrics, Stratified by Rehospitalization Status.

Fig 1 shows the steps taken to generate the study cohort. A total of 8,870 dialysis patients who met our inclusion criteria received KT between January 1, 2000 and December 31, 2010, of whom 8,788 (99%) survived their transplant hospitalization and were discharged. Of those KT recipients that were discharged from their transplant admission, 2543 (29%) experienced early rehospitalization after KT. Patients in the cohort lived in diverse regions of the United States, with representation from 40 distinct states. The median age of study participants was 52 years (IQR 41, 60); 35% were black race and 63% were male. Further, 1,745 (19%) of all KT recipients died during the follow-up period. The distribution of Elixhauser comorbidities, dialysis PF score, and prior hospitalization frequency differed based on rehospitalization status (Fig 2). S1 Table shows the most common reasons for rehospitalization based on each health status metric. Within the total study cohort of KT recipients, 67% (n = 5,905) had >one Elixhauser comorbidity at the time of KT, and 28% (n = 2481) had > one recent hospitalization prior to KT. The median time from PF measurement to KT was 225 days (IQR 113, 551). S2 Table shows the frequency of the most common Elixhauser comorbidities, stratified by rehospitalization status.

Table 1 shows the distribution of health metrics and traditional risk factors, based on rehospitalization status. In bivariate analysis, patients with early rehospitalization had a greater median number of Elixhauser comorbidities (3 vs 2, p <0.001) and lower PF score (60 vs 65, p<0.001) compared to patients not rehospitalized. More rehospitalized patients had greater than one hospitalization in the year prior to KT (33% vs. 26%, p<0.001). Recipient factors associated with early rehospitalization at the p<0.05 level on bivariate analysis included oldest age group (7% vs 5%, p<0.001), black race (38% vs 33%, p<0.001), diabetes (37% vs 31%, p <0.001), previous transplant (12% vs 10%, p = <0.001), hepatitis C (7% vs 5%, p = 0.002), longer dialysis duration (3.7 vs 3.3 years, p<0.001), and longer waiting list duration (2.15 vs 2.03 years, p<0.001). Transplant factors that were significantly associated with rehospitalization at the bivariate level included longer transplant length of stay (7 vs 6 days, p<0.001), deceased donor KT (85% vs. 82%, p<0.001), expanded criteria donor KT (18% vs 14%, p<0.001), and delayed graft function (33% vs 21%, p<0.001).

**Table 1:** Factors Associated with Early Rehospitalization after Kidney Transplant.

N = 8,788	Rehospitalized	Not Rehospitalized	p-value
	n = 2,543	n = 6,245	
**Health Status Metrics**			
Physical Function Score*	60 (35–80)	65 (40–85)	< .001
Elixhauser Score**	3 (1–5)	2 (0–4)	< .001
Prior Hospitalizations**			< .001
**0**	875 (34%)	2,644 (42%)	
**1**	824 (32%)	2,002 (32%)	
**>1**	844 (33%)	1,599 (26%)	
**Recipient Characteristics**^†^			
**Age Category**			< .001
**<35**	316 (12%)	901 (14%)	
**35–49**	749 (29%)	1,904 (30%)	
**50–69**	1,307 (51%)	3,151 (50%)	
**70–90**	171 (7%)	289 (5%)	
**Race**			< .001
**White**	1,116 (44%)	2,785 (45%)	
**Black**	961 (38%)	2,072 (33%)	
**Hispanic**	384 (15%)	1,139 (18%)	
**Asian**	82 (3%)	249 (4%)	
**Education Status**			0.33
**No College**	1,277 (50%)	3,022 (48%)	
**Some College**	478 (19%)	1,204 (19%)	
**College Graduate**	361 (14%)	966 (15%)	
**Unknown Status**	427 (17%)	1,053 (17%)	
**Female Gender**	943 (37%)	2,320 (37%)	0.96
**Previous Transplant**	293 (12%)	602 (10%)	0.009
**Diabetes**	938 (37%)	1,924 (31%)	< .001
**Obesity (BMI>30 kg/m2)**	744 (29%)	1,703 (27%)	0.06
**Missing BMI**	318 (13%)	780 (12%)	1
**Positive HCV Serostatus**	176 (7%)	327 (5%)	0.002
**Median Years on Dialysis**	3.76 (2.25–5.62)	3.33 (2.00–5.07)	< .001
**Median Waitlist Time (years)**	2.15 (1.05–3.52)	2.03 (0.99–3.27)	< .001
**Transplant LOS (days)**	7 (5–10)	6 (5–8)	< .001
**Donor/Allograft Factors**^†^			
**Deceased Donor**	2,153 (85%)	5,094 (82%)	< .001
**Expanded Criteria Donor**	453 (18%)	849 (14%)	< .001
**Delayed Graft Function**	842 (33%)	1,293 (21%)	< .001
Transplant Center Factors**			
Low Center Volume***	1,848 (73%)	4,627 (74%)	0.17
**Weekend Discharge**	456 (18%)	1,204 (19%)	0.15

All continuous variables expressed as median (IQR)

* Fresenius data, range 0 (lowest functioning)-100 (highest functioning)

** Medicare data

† OPTN Data

***defined as centers performing <150 kidney transplants on average per year

Abbreviation: BMI–Body Mass Index; LOS–length of stay

### Comparison of Adjusted Models

**Fig 3:**
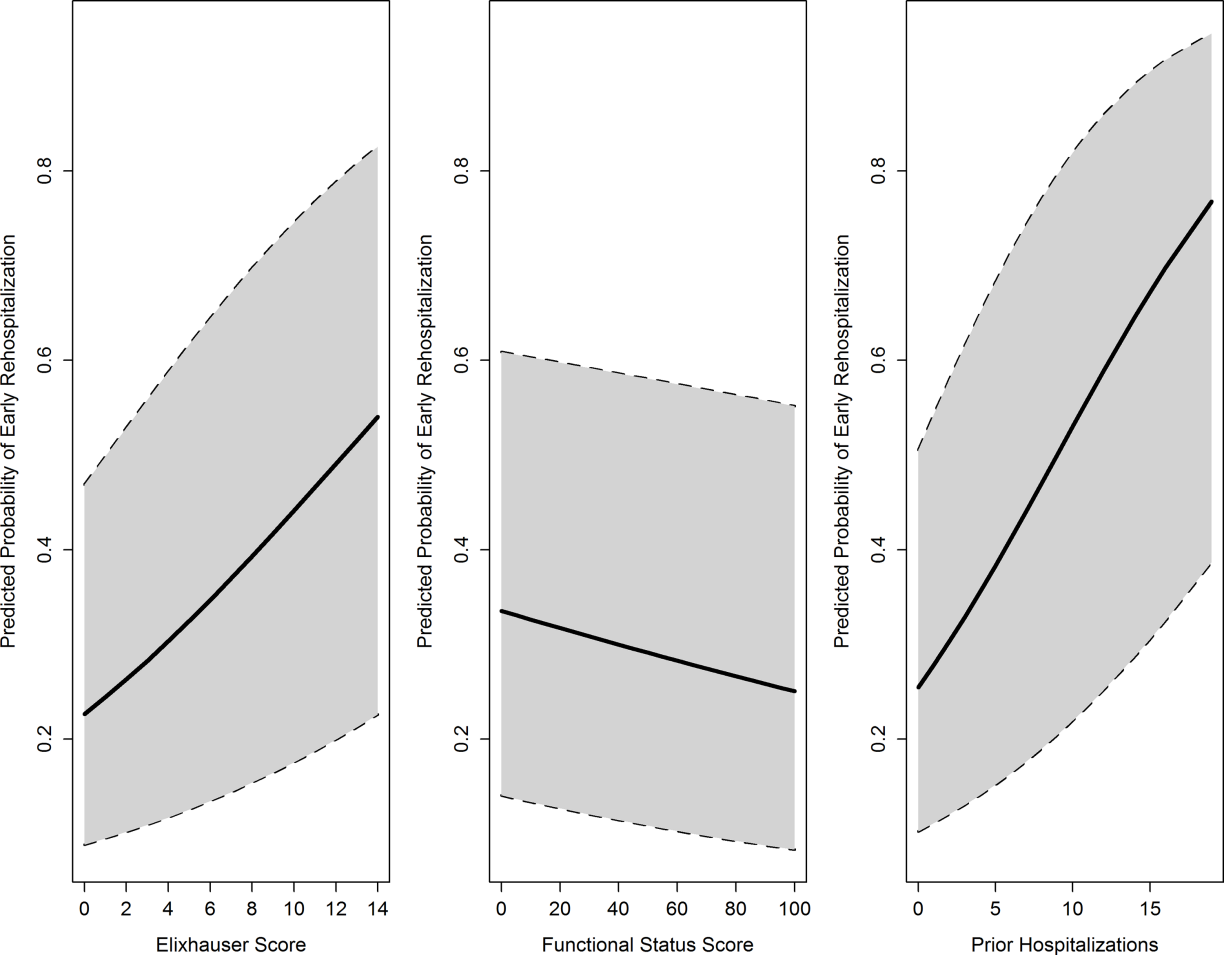
Adjusted Probability (with 95% Confidence Intervals) of Rehospitalization Based on Pre-Transplant Health Metrics. Adjusted for (1) *recipient* age category at transplant, sex, race, hepatitis C serostatus, obesity by body mass index (≥30 kg/m^2^), dialysis vintage (years), time on the waitlist (years), history of diabetes, history of previous solid organ transplant, education status, (2) *donor* type (live vs. deceased donor, expanded criteria deceased [ECD] donor); (3) *allograft* variables of delayed graft function, and (4) *process-of-care* variables of length of initial transplant hospitalization (days), weekend discharge (defined as discharge on Saturday or Sunday), and low transplant center volume (defined as <150 kidney transplants performed, on average, per year).

Table 2 illustrates the results of our modeling approach for the outcome of early rehospitalization. First, we tested the health metrics by adding each individually to the baseline multivariable model (models 1, 2, and 3, respectively). Fig 3 illustrates the probability of rehospitalization based on each metric, adjusted for all other baseline variables. Compared to the baseline model, the addition of any of the three health metrics improved model fit by AIC criterion. Likelihood ratio statistics demonstrated significant improvement in model fit of each model compared to the baseline model.

**Table 2:** Comparison of Logistic Regression Models for Early Rehospitalization.

N = 8,788	Base Model*	Model 1	Model 2	Model 3	Model 4	Model 5	Model 6	Model 7
**Elixhauser Score (OR per diagnosis)**		1.10 (1.08–1.13)^1^			1.10 (1.08–1.12)^1^	1.09 (1.07–1.12)^1^		1.09 (1.07–1.11)^1^
**Physical Function**^♯^								
**Second HighestQuartile**			1.16 (1.01–1.33)^3^		1.14 (0.99–1.30)		1.14 (0.99–1.31)	1.12 (0.98–1.29)
**Second LowestQuartile**			1.16 (1.02–1.33)^3^		1.11 (0.97–1.27)		1.12 (0.98–1.29)	1.09 (0.95–1.25)
**Lowest Quartile**			1.38 (1.20–1.59)^1^		1.28 (1.11–1.48)^1^		1.31 (1.14–1.51)^1^	1.24 (1.08–1.43)^2^
**Prior Hospitalizations(ref = none)**								
**1**				1.20 (1.07–1.34)^2^		1.16 (1.03–1.30)^3^	1.19 (1.06–1.33)^2^	1.16 (1.03–1.30)^3^
**>1**				1.49 (1.32–1.67)^1^		1.34 (1.19–1.51)^1^	1.45 (1.29–1.63)^1^	1.32 (1.17–1.49)^1^
**Summary Statistics**								
**AIC**	10,342	10,256	10,327	10,301	10,250	10,236	10,292	10,233
LR test p-value**		<0.001	<0.001	<0.001	<0.001	<0.001	<0.001	<0.001
-2*Log-likelihood (DF)	10,298 (21)	10,210 (22)	10,277 (24)	10,253 (23)	10,198 (25)	10,186 (24)	10,238 (26)	10,177 (27)
**C-Statistic**	0.611	0.626	0.615	0.619	0.628	0.629	0.622	0.631

Abbreviations: AIC—Akaike Information Criterion; LR–likelihood ratio; OR—Odds Ratio; DF—Degrees of Freedom

*Base Model contains recipient age, race, sex, education, diabetes status, hepatitis C status, obesity, years on dialysis, prior transplant status, deceased donor transplant, expanded criteria donor status, delayed graft function, transplant length of stay, waitlist time in years, weekend discharge, and low center volume.

**Compared to nested base model AIC

^♯^ Reference category: Highest Physical Function Quartile

^1^ p<0.001

^2^ p<0.01

^3^ p<0.05

**Fig 4:**
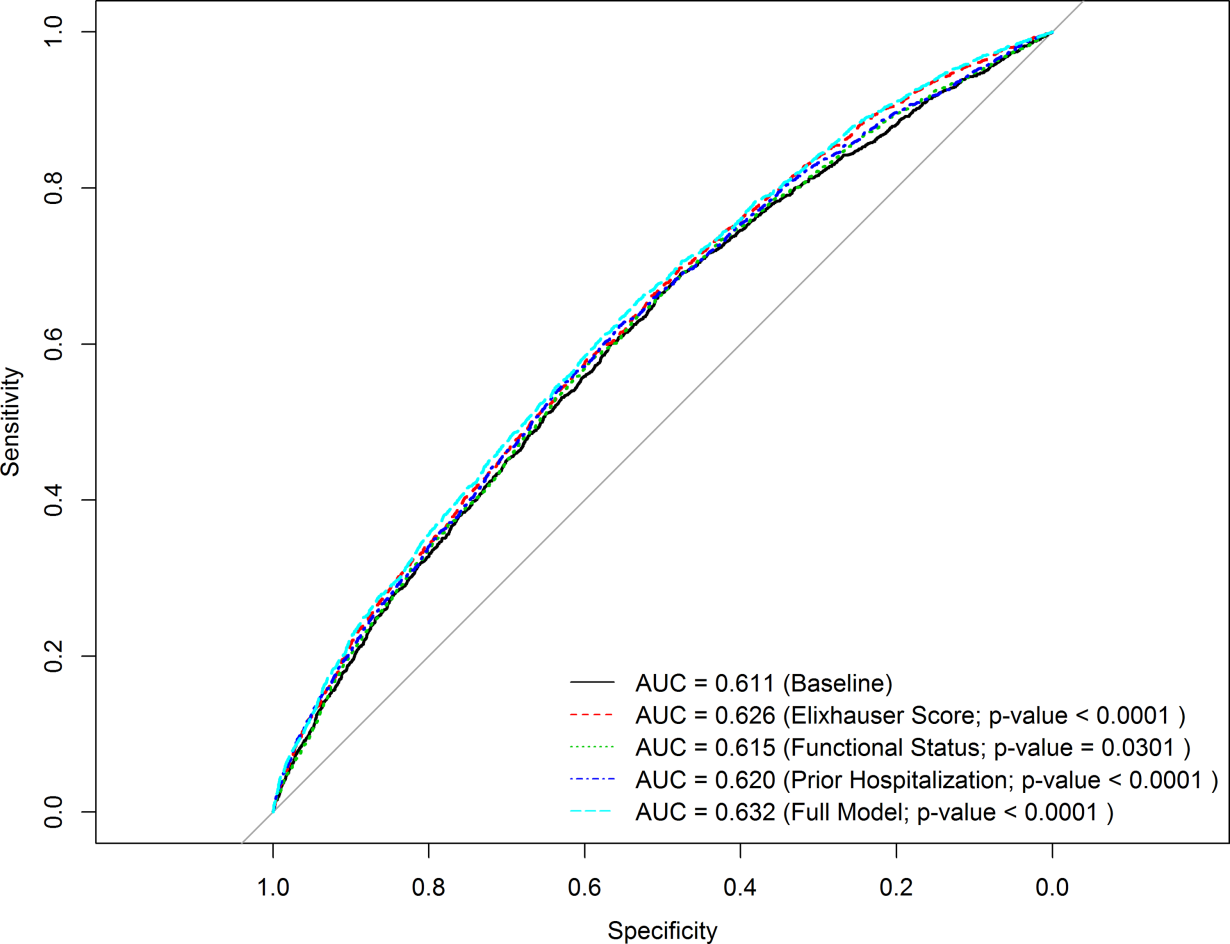
Prediction of the outcome of early rehospitalization after kidney transplantation using global health metrics. Baseline and subsequent logistic models adjusted for (1) *recipient* age category at transplant, sex, race, hepatitis C serostatus, obesity by body mass index (≥30 kg/m^2^), dialysis vintage (years), time on the waitlist (years), history of diabetes, history of previous solid organ transplant, education status, (2) *donor* type (live vs. deceased donor, expanded criteria deceased [ECD] donor); (3) *allograft* variables of delayed graft function, and (4) *process-of-care* variables of length of initial transplant hospitalization (days), weekend discharge (defined as discharge on Saturday or Sunday), and low transplant center volume (defined as <150 kidney transplants performed, on average, per year).

The optimal model by AIC criterion (i.e., lowest AIC) was the fully adjusted model (model 7). In the fully adjusted model, compared to those KT recipients with no pre-transplant hospitalizations, those with one hospitalization in the 12 months prior to KT had a 16% greater odds of early rehospitalization after KT (adjusted odds ratio [aOR] 1.16, 95% CI 1.03–1.30), and those with >1 hospitalization had a 32% greater odds of rehospitalization (aOR 1.32, 95% CI 1.17–1.49). Each additional pre-transplant Elixhauser comorbidity conferred a 9% increased odds of early rehospitalization (aOR 1.09, 95% CI 1.07–1.11). Compared to those within the highest PF quartile, KT recipients with the lowest PF quartile were also at higher risk for rehospitalization (aOR 1.24, 95% CI 1.08–1.43). All VIFs were <2 in the fully adjusted rehospitalization model, consistent with adequate levels independence between the predictors of interest [55]. Results were similar in sensitivity analyses in which patients with missing data on body mass index and education status, respectively, were assigned to the highest and lowest categories. Fig 4 shows the result of our comparison of the predictive ability of the health metrics under study, compared to the baseline model of recipient, donor, and process-of-care variables. Compared to the baseline model, with a c-statistic of 0.611, the fully adjusted model (baseline + 3 health metrics) modestly improved the c-statistic by 0.02 (new c-statistic 0.631, p<0.001).

### Secondary Analysis–Composite Outcome of Early Mortality and Early Rehospitalization

In our logistic regression models of the composite early mortality or rehospitalization endpoint, we found associations similar to those described in our primary analysis results between global health metrics and the risk of early rehospitalization alone (see S3 Table).

### Secondary Analysis–Post-Transplant Mortality

Table 3 demonstrates the results of our modeling approach for the outcome of post-transplant mortality. In order to determine the degree to which pre-transplant health metrics attenuated the association of early rehospitalization with post-transplant mortality, early rehospitalization was modeled as a time-dependent covariate in consecutive Cox regression models for post-transplant mortality that were also adjusted for the three global health metrics and numerous traditional risk factors (see S4 Table for data on the distribution of covariates based on mortality status).

**Table 3:** Association of Early Rehospitalization and Post-Transplant Mortality in Cox Proportional Hazards Models, with Adjustment for Pre-Transplant Health Status.

N = 8,870	Model A (HR, 95% CI)	Model B ^♯^	Model C	Model D	Model E	Model F	Model G	Model H	Model I	Full Model
**Early Rehospitalization**	1.64 (1.49–1.81)^1^		1.48 (1.34–1.63)^1^	1.43 (1.29–1.58)^1^	1.46 (1.32–1.61)^1^	1.45 (1.31–1.60)^1^	1.42 (1.28–1.57)^1^	1.42 (1.28–1.56)^1^	1.44 (1.30–1.59)^1^	1.41 (1.28–1.56)^1^
**Health Status Metrics**										
**Elixhauser Score (per comorbidity)**				1.07 (1.05–1.09)^1^			1.06 (1.04–1.08)^1^	1.05 (1.03–1.07)^1^		1.05 (1.03–1.07)^1^
**Physical Function Quartile (ref = highest)**										
**Second Highest**					1.11 (0.95–1.29)		1.10 (0.94–1.28)		1.08 (0.93–1.26)	1.07 (0.92–1.25)
**Second Lowest**					1.29 (1.11–1.48)^1^		1.25 (1.08–1.44)^2^		1.23 (1.06–1.43)^2^	1.21 (1.05–1.40)^2^
**Lowest**					1.65 (1.43–1.90)^1^		1.57 (1.36–1.82)^1^		1.56 (1.35–1.80)^1^	1.52 (1.31–1.75)^1^
**Prior Hospitalization (ref = none)**										
**1**						1.10 (0.98–1.24)		1.07 (0.95–1.20)	1.09 (0.96–1.22)	1.06 (0.94–1.19)
**>1**						1.50 (1.33–1.68)^1^		1.39 (1.24–1.57)^1^	1.43 (1.27–1.60)^1^	1.35 (1.20–1.51)^1^
**Summary Statistics**										
**AIC**	31,263	30,546	30,490	30,450	30,438	30,443	30,409	30,421	30,402	30,386
Likelihood Ratio p-value*				<0.001	<0.001	<0.001	<0.001	<0.001	<0.001	<0.001
-2*Log-likelihood (DF)	31,260 (1)	30,504 (21)	30,446 (22)	30,404 (23)	30,388 (25)	30,396 (24)	30,356 (26)	30,370 (25)	30,348 (27)	30,330 (28)
**C-Statistic**^+^	0.560	0.719	0.726	0.730	0.731	0.730	0.734	0.733	0.734	0.736

Abbreviations: HR—hazard ratio; CI—confidence interval; ref—reference; AIC—Akaike Information Criterion; DF—Degrees of Freedom

♯ Model B and subsequent models adjusted for recipient age, race, sex, education, diabetes status, obesity status, hepatitis C status, years on dialysis, prior transplant status, deceased donor transplant, expanded criteria donor status, delayed graft function, transplant length of stay, waitlist time in years, low center volume, and weekend discharge.

* Models D-I and full model compared to Model C

+ C-statistics calculated for each model, predicting post-transplant mortality

1 p<0.001

2 p<0.01

**Fig 5:**
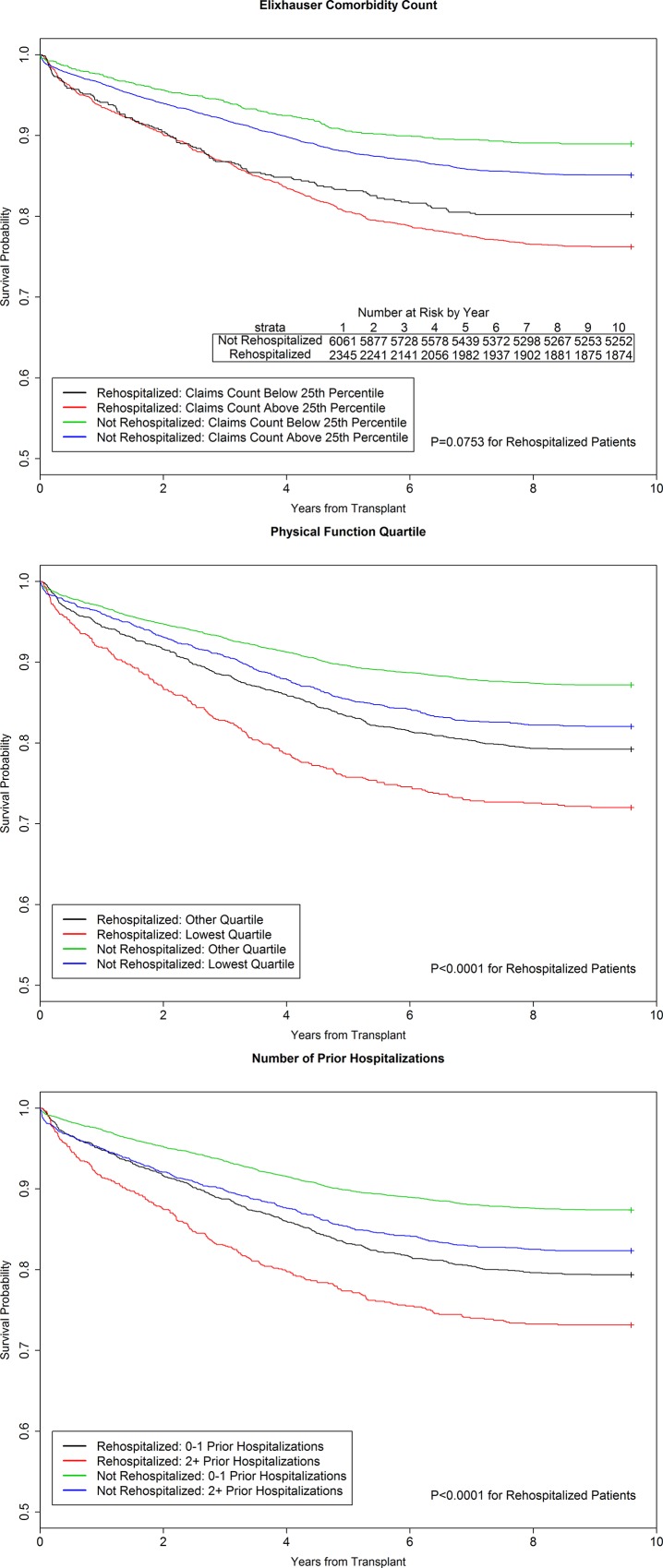
Poor Global Health Status and Early Rehospitalization Both Augment Mortality Risk after Kidney Transplantation. Cox models also adjusted for (1) *recipient* age category at transplant, sex, race, hepatitis C serostatus, obesity by body mass index (≥30 kg/m^2^), dialysis vintage (years), time on the waitlist (years), history of diabetes, history of previous solid organ transplant, education status, (2) *donor* type (live vs. deceased donor, expanded criteria deceased [ECD] donor); (3) *allograft* variables of delayed graft function, and (4) *process-of-care* variables of length of initial transplant hospitalization (days), weekend discharge (defined as discharge on Saturday or Sunday), and low transplant center volume (defined as <150 kidney transplants performed, on average, per year).

Early rehospitalization remained a strong predictor of mortality after KT after adjustment for recipient, donor, and center factors (adjusted Hazard Ratio [aHR] for rehospitalization 1.48, 95% CI 1.33–1.63). Further adjustment for pre-transplant health metrics modestly attenuated the magnitude of the association of early rehospitalization and post-transplant mortality (aHR for rehospitalization after adjustment for health metrics: 1.41, 95% CI 1.28–1.56). All three pre-transplant health metrics were independently associated with post-KT mortality (see Fig 5 for survival curves). Adjustment for the three pre-transplant metrics modestly improved prediction of post-KT mortality over adjustment for rehospitalization and traditional risk factors alone (c-statistic 0.726 vs 0.736, p<0.001).

## Discussion

We investigated the ability of three commonly available metrics of pre-transplant global health status to serve as signals of early rehospitalization and mortality risk after KT. Our results demonstrated that after adjustment for numerous traditional recipient, donor, and process-of-care risk factors, the Elixhauser Comorbidity Index, pre-transplant PF, and pre-KT hospitalization frequency are independently associated with early rehospitalization after KT. Each metric of global health on dialysis may have a role in the transplant evaluation process, providing complementary information on KT candidates’ evolving health status as many wait for multiple years on dialysis for KT, and enabling transplant providers to risk-stratify kidney recipients as early as possible in the post-transplant course.

Additionally, consistent with prior studies, this study demonstrated that early rehospitalization after KT is an independent predictor of post-KT mortality [2, 8]. However, our study further revealed that global health metrics that are not included in traditional risk-adjustment algorithms but are routinely measured by dialysis providers and health systems are independently associated with post-transplant mortality, even after adjustment for early rehospitalization events. Notably, our study found that adjustment for all three health status metrics only modestly attenuated the association between early rehospitalization after KT and post-transplant mortality. Therefore, future studies should explore whether other potentially measurable and modifiable determinants of health status, such as poor social support, may also help to explain the observed association between early rehospitalization and post-transplant mortality. Comorbidity scores such as the Elixhauser Comorbidity Index have been used extensively to describe patient burdens of disease, allowing comparisons of outcomes across hospitals with diverse patient populations [20, 25, 56, 57]. Our study showed that the Elixhauser Comorbidity Index remained an independent predictor of early rehospitalization after KT even after adjustment for recipient, donor, allograft, and center factors that are unavailable in traditional claims data. The Elixhauser comorbidity diagnosis codes for anemia, hypertension, and diabetes were among the most common in our cohort, and every Elixhauser comorbidity occurred more frequently among rehospitalized versus never-rehospitalized kidney recipients. However, the highest proportional increases in codes among rehospitalized patients were for liver disease and coagulopathy, congestive heart failure, and peripheral vascular disease, which are all plausibly on the causal pathways of previously identified common reasons for early rehospitalization after KT, including poor wound healing, volume overload and infection [2]. Our findings suggest that transplant providers with knowledge of their patients’ pre-KT medical claims data may be able to use these data for risk stratification post-KT. While many pre-KT comorbidities may be consequences of a patient’s end stage renal disease, future studies are needed to examine interventions that may improve post-KT outcomes (e.g., optimizing cardiovascular fitness pre-transplant for those with congestive heart failure, planning home visits or more frequent outpatient assessments for those with a high burden of pre-transplant comorbidities).

Pre-transplant PF might also help to explain rehospitalization events after KT. Functional impairment has been recently described as a risk factor for rehospitalization among older Medicare recipients [58]. We found that compared to KT candidates with the highest pre-transplant PF, those with the poorest function were significantly more likely to be rehospitalized early after KT. Our findings are consistent with prior studies that have observed that pre-transplant testing of PF may provide critical knowledge of KT candidate risk [7, 34, 35, 59]. Dialysis patients often suffer from poor global health [60], and two studies among national cohorts of KT recipients have shown that poor functional status (self-reported through the PF SF-36) is strongly associated with mortality [34] and hospitalizations within six months post-transplant [35]. Ours is the first national study to demonstrate that self-reported PF while on dialysis, as measured by the SF-36, is independently associated with early rehospitalization after KT. Regular communication between dialysis and transplant providers on KT candidates, including information on PF while on dialysis, may help transplant providers prognosticate early post-KT hospitalizations. Future prospective studies are needed to identify strategies to optimize outcomes for KT candidates with poor PF, and investigate interventions, such as exercise programs [61–63], to improve KT candidates’ pre-transplant PF.

Finally, our results also demonstrated that knowledge of waitlisted patients’ health care utilization provides important insight into rehospitalization and mortality risk after KT. Patients with chronic kidney disease and end stage renal disease have high baseline rates of health care utilization: a 2014 study of US Renal Data System (USRDS) data reported that 58.3% of in-center hemodialysis patients were hospitalized within one year of treatment and 81.8% of these were rehospitalized within the following year. Hospitalization patterns may remain consistent for some time post- KT [42], as recipient health status changes in response to improving kidney function. However, while KT often increases short-term health care utilization compared to dialysis, it also usually improves long-term outcomes, including quality of life and survival [64]. In our study, KT recipients who were hospitalized in the year prior to KT were more likely to be rehospitalized early after KT and were also more likely to die, suggesting that transplant centers that routinely ascertain hospitalization events for waitlisted candidates may be able to utilize these data for risk-stratification in the early post-transplant period. Future studies should investigate whether high pre-transplant health care utilization among kidney candidates is associated with modifiable factors such as inadequate transportation, psychosocial barriers, or limitations in health literacy or numeracy.

Our study must be considered with respect to its limitations. Concerns may arise about the generalizability of our findings to those KT recipients who are not Medicare beneficiaries, or not on dialysis. For example, our study may not be generalizable to KT recipients who received less than one year of maintenance dialysis prior to KT. However, most KT recipients receive some dialysis before transplant, and for many, long waiting times necessitate prolonged dialysis exposure, which is strongly associated with worsening health status [27, 65]. Our study sample was also diverse and nationally representative, as Fresenius Medical Care provided dialysis services to one third of all dialysis patients in the US during the study period [16, 38], such that 40 of the 50 US states were represented in the cohort. Also, as the traditional 30 day rehospitalization metric may introduce bias because it only includes those patients that achieved discharge from their index hospitalization (i.e., transplant hospitalization in our study), we performed a secondary analysis that included those recipients that died during their index admission in a composite outcome of early death or readmission, and found similar associations with global health metrics. Another potential limitation of our study is the lack of granular data on reasons for rehospitalization. The most frequent readmission codes encountered in our cohort did not substantially differ based on pre-transplant health metrics (S1 Table), but Medicare claims do not offer detailed descriptors that may elucidate whether a readmission was potentially avoidable, for example.

We also note that while the explanatory models revealed significant associations between global health metrics and rehospitalization, all models had limited predictive ability as assessed by the c-statistic (e.g., the fully adjusted rehospitalization model c-statistic was 0.63). Inclusion of health metrics improved our c-statistic for predicting rehospitalization by 0.02 (after adjusting for recipient, donor, and process-of-care covariates). Interestingly, studies seeking to predict rehospitalization events in large, general medicine populations have yielded similarly modest discriminative ability, with the majority of c-statistics ranging from 0.60 to 0.72 [37, 66–69]. Predictive modeling of early rehospitalization, particularly after KT, presents a challenge likely because the etiology of these events is often multifactorial and diverse [2]. Some KT recipients may be rehospitalized because of post-transplant fluid collections, while others may have insufficient social support, while others may experience problems related to miscommunication about medications at discharge. KT recipients face numerous physiologic challenges during transplant admissions, including surgical wounds, massive volume shifts and new complex medication regimens with many potential side effects. An individual’s early response to some of these challenges may not be easily predicted, even when global health status is measured. Future studies that seek to derive and validate prediction models for kidney transplant rehospitalization may consider exploring additional drivers of health status, including metrics of health literacy, numeracy, and social support, to improve discrimination.

In conclusion, early rehospitalization after KT is a common event and a predictor of death after transplant, and transplant providers are in need of metrics to better understand the medical complexity leading to these events. We studied three potential metrics among dialysis patients who received KT: the Elixhauser Comorbidity Index, pre-transplant PF, and frequency of pre-transplant hospitalizations. We found that each metric is independently associated with early rehospitalization events after KT, and that evidence of poor global health at the time of KT signals heightened mortality risk after KT. Additionally, even after adjustment for global health metrics, early rehospitalization remains strongly associated with post-transplant mortality. Health care providers with access to these metrics of global health status may be able to utilize these data to identify the kidney recipients most at risk for rehospitalization and other adverse events after transplant, providing alternative strategies during waiting time and immediately after KT to optimize post-transplant outcomes.

## Supporting Information

S1 Table Most Frequent Reasons for Rehospitalization, Stratified by Health Status Metric. (DOCX)

S2 Table Most Frequent Pre-Transplant Elixhauser Comorbidities, Stratified by Hospitalization Status. (DOCX)

S3 Table Comparison of Logistic Regression Models for the Composite Outcome of Early Rehospitalization or Death within 30 days of Discharge from Kidney Transplantation. (DOCX)

S4 Table Factors Associated with Mortality after Kidney Transplant. (DOCX)
