# Supplementary material for: Measures of Global Health Status on Dialysis Signal Early Rehospitalization Risk after Kidney Transplantation
Source: PLoS One. 2016 Jun 3;11(6):e0156532. doi: 10.1371/journal.pone.0156532 (PMC4892690; doi:10.1371/journal.pone.0156532)
Supplement: S1 Table — (DOCX) [file pone.0156532.s001.docx]

**S1 Table: Most Frequent Reasons for Rehospitalization, Stratified by Health Status Metric**

| **Diagnosis** | **Rank, Total Cohort**  **(n=2,543)** | **Highest PF Quartile** | **Other PF Quartile** | **0 Prior Hospitalizations** | **>1 Prior Hospitalization** | **0 Elixhauser Comorbidities** | **>1 Elixhauser Comorbidities** |
| --- | --- | --- | --- | --- | --- | --- | --- |
| **Complications of Transplanted Kidney** | 1 | 1 | 1 | 1 | 1 | 1 | 1 |
| **Other Postoperative Infection** | 2 | 2 | 2 | 3 | 2 | 2 | 2 |
| **Hyperpotassemia** | 3 | 3 | 4 | 2 | 3 | 4 | 3 |
| **Urinary Tract Infection, site not specified** | 4 | 4 | 3 | 4 | 4 | 3 | 4 |
| **Congestive Heart Failure, Unspecified** | 5 | 5 |  |  | 5 |  |  |
| **Volume Depletion** | 6 |  | 5 | 5 |  |  | 5 |
| **Hematoma Complicating a Procedure** | 7 |  |  |  |  | 5 |  |
| Abbreviations: PF—Physical Function | | | | | | | |
