# Supplementary material for: Measures of Global Health Status on Dialysis Signal Early Rehospitalization Risk after Kidney Transplantation
Source: PLoS One. 2016 Jun 3;11(6):e0156532. doi: 10.1371/journal.pone.0156532 (PMC4892690; doi:10.1371/journal.pone.0156532)
Supplement: S2 Table — (DOCX) [file pone.0156532.s002.docx]

| **Comorbidity** | **% Total Cohort, n=8,788** | **% Not Rehospitalized, n=6,245**  **(Column 1)** | **% Rehospitalized, n=2,543**  **(Column 2)** | **Relative % Increase Among Rehospitalized*** |
| --- | --- | --- | --- | --- |
| **Anemia** | 65.1 | 62.2 | 72.2 | 16.0 |
| **Hypertension**** | 58.0 | 55.6 | 63.7 | 14.5 |
| **Diabetes (Type I and II)**** | 30.8 | 28.4 | 36.5 | 28.3 |
| **Fluid and electrolytes disorders** | 27.7 | 26.2 | 31.4 | 19.7 |
| **Liver disease / Coagulopathy** | 14.2 | 12.8 | 17.6 | 37.7 |
| **Congestive heart failure** | 12.3 | 11.0 | 15.5 | 40.6 |
| **Peripheral vascular disease** | 12.3 | 11.2 | 15.1 | 34.5 |
| **Valvular Heart Disease** | 10.0 | 9.4 | 11.3 | 19.9 |
| **Hypothyroidism** | 6.9 | 6.3 | 8.3 | 32.2 |
| **Chronic lung disease** | 6.1 | 5.5 | 7.4 | 34.1 |
| *(Column 2/ Column 1) - 100  **Combined category of complicated and uncomplicated | | | | |

**S2 Table: Most Frequent Pre-Transplant Elixhauser Comorbidities, Stratified by Hospitalization Status.**
