# Supplementary material for: Measures of Global Health Status on Dialysis Signal Early Rehospitalization Risk after Kidney Transplantation
Source: PLoS One. 2016 Jun 3;11(6):e0156532. doi: 10.1371/journal.pone.0156532 (PMC4892690; doi:10.1371/journal.pone.0156532)
Supplement: S3 Table — (DOCX) [file pone.0156532.s003.docx]

**S3 Table: Comparison of Logistic Regression Models for the Composite Outcome of Early Rehospitalization or Death within 30 days of Discharge from Kidney Transplantation**

| N=8.870 | **Base Model B*** | **Model 1b** | **Model 2b** | **Model 3b** | **Model 4b** | **Model 5b** | **Model 6b** | **Model 7b** |
| --- | --- | --- | --- | --- | --- | --- | --- | --- |
| **Elixhauser Score (OR per diagnosis)** |  | 1.11 (1.09-1.13)^1^ |  |  | 1.10 (1.08-1.13)^1^ | 1.09 (1.07-1.12)^1^ |  | 1.09 (1.07-1.11)^1^ |
|  |  |  |  |  |  |  |  |  |
| **Physical Function^♯^** |  |  |  |  |  |  |  |  |
| **Second Highest Quartile** |  |  | 1.15 (1.00-1.31)^3^ |  | 1.12 (0.98-1.28) |  | 1.12 (0.98-1.29) | 1.10 (0.96-1.27) |
| **Second Lowest Quartile** |  |  | 1.16 (1.01-1.32)^3^ |  | 1.10 (0.97-1.26) |  | 1.12 (0.98-1.27) | 1.08 (0.94-1.23) |
| **Lowest Quartile** |  |  | 1.38 (1.20-1.58)^1^ |  | 1.27 (1.11-1.46)^1^ |  | 1.30 (1.13-1.50)^1^ | 1.23 (1.07-1.42)^2^ |
|  |  |  |  |  |  |  |  |  |
| **Prior Hospitalizations**  **(ref=0)** |  |  |  |  |  |  |  |  |
| **1** |  |  |  | 1.20 (1.07-1.34)^2^ |  | 1.16 (1.03-1.30)^3^ | 1.19 (1.06-1.33)^2^ | 1.15 (1.03-1.29)^3^ |
| **>1** |  |  |  | 1.52 (1.35-1.70)^1^ |  | 1.36 (1.21-1.53)^1^ | 1.48 (1.32-1.66)^1^ | 1.34 (1.19-1.51)^1^ |
| **Summary Statistics** | | | | | | | | |
| **AIC** | 10,532 | 10,438 | 10,517 | 10,484 | 10,433 | 10,415 | 10,477 | 10,413 |
| **LR test p-value**** |  | <0.001 | <0.001 | <0.001 | <0.001 | <0.001 | <0.001 | <0.001 |
| **-2*Log-likelihood (DF)** | 10,492 (19) | 10,396 (20) | 10,471 (22) | 10,440 (21) | 10,385 (23) | 10,369 (22) | 10,427 (24) | 10,361 (25) |
| **C-Statistic** | 0.616 | 0.631 | 0.619 | 0.625 | 0.633 | 0.635 | 0.627 | 0.636 |
| Abbreviations: AIC—Akaike Information Criterion; LR –likelihood ratio; OR—Odds Ratio; DF—Degrees of Freedom  *Base Model contains recipient age, race, sex, education, diabetes status, hepatitis C status, obesity, years on dialysis, prior transplant status, deceased donor transplant, expanded criteria donor status, delayed graft function, transplant length of stay, waitlist time in years, weekend discharge, and low center volume.  **Compared to nested base model AIC  ^♯^ Reference category: Highest Physical Function Quartile  ^1^ p<0.001  ^2^ p<0.01  ^3^ p<0.05 | | | | | | | | |
