# Supplementary material for: Measures of Global Health Status on Dialysis Signal Early Rehospitalization Risk after Kidney Transplantation
Source: PLoS One. 2016 Jun 3;11(6):e0156532. doi: 10.1371/journal.pone.0156532 (PMC4892690; doi:10.1371/journal.pone.0156532)
Supplement: S4 Table — (DOCX) [file pone.0156532.s004.docx]

**S4 Table:** **Factors Associated with Mortality after Kidney Transplant.**

| **N=8,870** | **Died During Follow-Up** | **Alive During Follow-Up** | **p-value** |
| --- | --- | --- | --- |
|  | n=1,745 | n=7,125 |  |
|  |  |  |  |
| **Rehospitalized Early** | 669 (38%) | 1,874 (26%) | <.001 |
|  | | | |
| **Health Status Metrics** | | | |
| **Physical Function Score*** | 50 (30-75) | 65 (45-85) | <.001 |
| **Elixhauser Score**** | 4 (2-5) | 2 (0-4) | <.001 |
| **Prior Hospitalizations**** |  |  | <.001 |
| **0** | 592 (34%) | 2,949 (41%) |  |
| **1** | 537 (31%) | 2,311 (32%) |  |
| **>1** | 616 (35%) | 1,865 (26%) |  |
| **Recipient Characteristics^†^** | | | |
| **Age Category** |  |  | <.001 |
| **<35** | 99 (6%) | 1,122 (16%) |  |
| **35-49** | 351 (20%) | 2,318 (33%) |  |
| **50-69** | 1,129 (65%) | 3,387 (48%) |  |
| **70-90** | 166 (10%) | 298 (4%) |  |
| **Race** |  |  | <.001 |
| **White** | 937 (54%) | 2,999 (42%) |  |
| **Black** | 550 (32%) | 2,513 (35%) |  |
| **Hispanic** | 207 (12%) | 1,328 (19%) |  |
| **Asian** | 51 (3%) | 285 (4%) |  |
| **Education Status** |  |  | 0.05 |
| **No College** | 902 (52%) | 3,444 (48%) |  |
| **Some College** | 321 (18%) | 1,372 (19%) |  |
| **College Graduate** | 235 (13%) | 1,105 (16%) |  |
| **Unknown Status** | 287 (16%) | 1,204 (17%) |  |
| **Female Gender** | 611 (35%) | 2,683 (38%) | 0.04 |
| **Previous Transplant** | 157 (9%) | 748 (10%) | 0.06 |
| **Diabetes** | 831 (48%) | 2,075 (29%) | <.001 |
| **Obesity (BMI>30 kg/m2)** | 489 (28%) | 1,989 (28%) | 0.93 |
| **Missing BMI** | 234 (13%) | 882 (12%) | 0.24 |
| **Positive HCV Serostatus** | 165 (9%) | 346 (5%) | <.001 |
| **Median Years on Dialysis** | 3.23 (1.92-5.04) | 3.50 (2.10-5.29) | <.001 |
| **Median Waitlist Time (years)** | 1.58 (0.75-2.75) | 2.17 (1.09-3.46) | <.001 |
| **Transplant LOS (days)** | 7 (5-11) | 6 (5-8) | <.001 |
| **Donor/Allograft Factors^†^** | | | |
| **Deceased Donor** | 1,482 (85%) | 5,840 (82%) | 0.003 |
| **Expanded Criteria Donor** | 347 (20%) | 972 (14%) | <.001 |
| **Delayed Graft Function** | 569 (33%) | 1,604 (23%) | <.001 |
| **Transplant Center Factors**** | | | |
| **Low Center Volume***** | 1,321 (76%) | 5,218 (73%) | 0.04 |
| **Weekend Discharge** | 331 (19%) | 1,349 (19%) | 0.97 |
| All continuous variables expressed as median (IQR)  * Fresenius data, range 0 (lowest functioning)-100 (highest functioning); ** Medicare data; **^†^** OPTN Data  ***defined as centers performing <150 kidney transplants on average per year  Abbreviation: BMI – Body Mass Index; LOS – length of stay | | | |
